# Molecular identification of trypanosome species in trypanotolerant cattle from the south of Gabon

**DOI:** 10.1051/parasite/2017003

**Published:** 2017-02-01

**Authors:** Gaël Darren Maganga, Jacques-François Mavoungou, Nadine N’dilimabaka, Ivan Cyr Moussadji Kinga, Bertrand Mvé-Ondo, Illich Manfred Mombo, Barthélémy Ngoubangoye, Brieuc Cossic, Clency Sylde Mikala Okouyi, Alain Souza, Eric Maurice Leroy, Brice Kumulungui, Benjamin Ollomo

**Affiliations:** 1 Centre International de Recherches Médicales de Franceville BP 769 Franceville Gabon; 2 Université des Sciences et Techniques de Masuku (USTM), Institut National Supérieur d’Agronomie et de Biotechnologies (INSAB) BP 913 Franceville Gabon; 3 Institut de Recherche en Écologie Tropicale (IRET-CENAREST) BP 13354 Libreville Gabon; 4 SIAT-Gabon Group BP 3928 Libreville Gabon

**Keywords:** *Trypanosoma congolense*, *Trypanosoma vivax*, Trypanotolerant cattle, *ITS1* PCR

## Abstract

The aim of this study was to provide information on trypanosome species infecting trypanotolerant cattle from southern Gabon. The study was conducted on 224 trypanotolerant cattle from three regions located in southern Gabon, using *ITS1* primer-based PCR. Seventy-two (32%) N’dama cattle were found polymerase chain reaction (PCR) positive with trypanosomes. The overall prevalence of trypanosomosis was 57% (63/110), 4% (4/100), and 36% (5/14) in the Gala section of the Nyanga ranch, the Miyama ranch, and Ossiele, respectively. *Trypanosoma congolense* and *Trypanosoma vivax* were identified. In Gala section and Ossiele, *T. congolense* and *T. vivax* were found. In the Miyama ranch, only *T. vivax* was identified. Mixed infections were also found. The *forest* (9%) and *savannah* (63%) subgroups of *T. congolense* were identified. The presence of the two subgroups was detected in 16 out of 56 cattle (29%). *T. congolense* and *T. vivax* would appear to be the main agents responsible for bovine trypanosomosis in southern Gabon. Although trypanotolerant, N’dama cattle may serve as a reservoir, and this should be further studied. On the other hand, these trypanotolerant cattle can be reared in such tsetse infested areas, which gives them an advantage compared to other trypanosensitive breeds, and this shows that they represent a key factor in biodiversity which has to be promoted.

## Abstract (fr)

Le but de cette étude était de fournir des informations sur les espèces de trypanosomes infectant les bovins trypanotolérants du sud du Gabon. L’étude a été menée sur 224 bovins trypanotolérants, de trois régions situées dans le sud du Gabon, en utilisant la PCR basée sur les amorces *ITS1*. Soixante-douze (32 %) bovins N’Dama ont été trouvés positifs en PCR pour des trypanosomes. La prévalence globale de la trypanosomose était respectivement 57 % (63/110), 4 % (4/100) et 36 % (5/14) dans la section Gala du ranch Nyanga, au ranch Miyama et à Ossiele. *Trypanosoma congolense* et *Trypanosoma vivax* ont été identifiés. Dans la section Gala et à Ossiele, *T. congolense* et *T. vivax* ont été trouvés. Au ranch Miyama, seul *T. vivax* a été identifié. Des infections mixtes ont également été trouvées. Les sous-groupes forêt (9 %) et savane (63 %) de *T. congolense* ont été identifiés. Les deux sous-groupes ont été détectés chez 16 des 56 bovins (29 %). *T. congolense* et *T. vivax* pourraient être les principaux agents responsables de la trypanosomose bovine dans le sud du Gabon. Bien que trypanotolérants, les bovins N’Dama pourraient servir de réservoir, et cela devrait être davantage étudié. D’un autre côté, ces bovins trypanotolérants peuvent être élevés dans des zones infestées de glossines, ce qui leur confère un avantage certain par rapport aux races trypanosensibles, et cela montre qu’ils jouent un rôle clé dans la biodiversité, qui doit être promu.

## Introduction

Trypanosomes are extracellular protozoan parasites of vertebrates, including domestic cattle, from tropical regions. Trypanosomes cause diseases known as trypanosomosis. In sub-Saharan Africa, cattle trypanosomosis is caused mainly by *Trypanosoma congolense*, *Trypanosoma brucei*, and *Trypanosoma vivax* (responsible for the disease known as *nagana* in cattle). African mammals harbor other pathogenic trypanosomes such as *Trypanosoma simiae*, *Trypanosoma suis*, *Trypanosoma uniforme*, *Trypanosoma evansi* (responsible for *surra*, trypanosomosis of Camelidae and Equidae), and *Trypanosoma equiperdum* (responsible for dourine, equine trypanosomosis) [30]. Trypanosomes are mainly transmitted between mammalian hosts by the bite of an infected tsetse fly (biological vectors); however, some trypanosomes can be transmitted by hematophagous Diptera (mechanical vectors) belonging to the Tabanidae, Stomoxyinae, and Hippoboscidae [10, 19, 26]. This mechanical transmission is especially known for *T. vivax* and *T. evansi*, which are mainly found in areas beyond the range of tsetse flies [6, 10].

In Gabon, the determination of breeding areas and the introduction of animal herds are problematic in terms of animal trypanosomosis. The few savannah areas of southern Gabon are crossed by many forest galleries and dotted with dense groves. These woodlands are the preferred shelters of tsetse that thrive in this type of environment. In addition, only little information is available on the status of bovine trypanosomosis in Gabon, especially regarding trypanosomes and tsetse species as well as the affected geographical areas. However, the presence of *Glossina haningtoni* and *Glossina tabaniformis* has been confirmed in forests and forest galleries of the Nyanga valley, southern Gabon [29]. A survey conducted in 1986 in the Okouma ranch, in the south-east, revealed the presence of *G. nashi* and *G. tabaniformis* [14]. *G. palpalis palpalis* was identified in different regions of the country: in the region of the Nyanga valley [29], as the main species, and during active outbreaks of human trypanosomosis, in Komo Mondah and Bendjé, in the north [11, 12], and in the north-east (Ogooué-Ivindo province) [32]. In this last region, *G. frezili*, *G. fuscipes fuscipes*, *G. nashi*, and *G. fusca congolense* have also been identified [32]. In the Nyanga valley, *Trypanosoma congolense* and *Trypanosoma vivax* have been identified in *G. p. palpalis*. During the Komo Mondah and Bendjé outbreaks, entomological research revealed infection of tsetse by *T. brucei*, *T. vivax*, and *T. congolense*. The prevalence and incidence of bovine trypanosomosis in Gabon are very poorly known. The few studies that have been conducted concern the Okouma ranch, south-east of Gabon. *Trypanosoma vivax* was identified in cattle at a rate of 26% using parasitological tools [14].

The aim of this study was to provide information on the circulation of trypanosome species in trypanotolerant cattle from southern Gabon and to determine the molecular prevalence of infection of these species in order to evaluate the risk of bovine trypanosomosis in the main cattle breeding regions of Gabon.

## Material and methods

### Study areas

**Figure 1.**
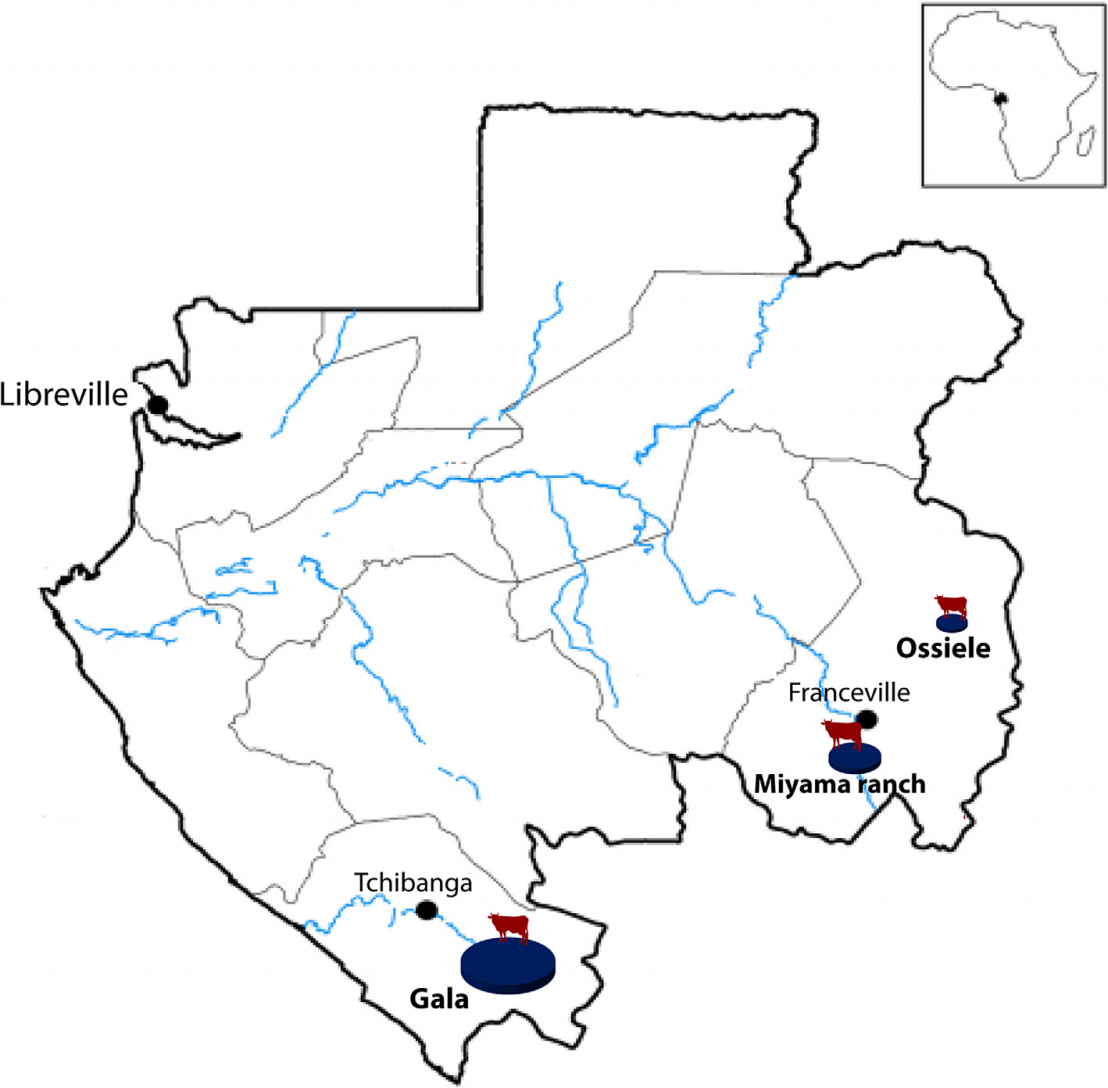
*Map of Gabon with the location of sampling sites*. The three study areas are shown with blue circles. The diameter of the circles is proportional to the numbers of animals bred in each area.

The study was carried out on cattle from three areas located in two provinces of the south-east of Gabon: the Miyama ranch and the rural town of Ossiele, in the Haut-Ogooué province; the Gala section of the Nyanga ranch in Nyanga province (Fig. 1). In addition to breeding cattle, these areas were selected because animal trypanosomosis has frequently been suspected on the basis of clinical signs.

The Miyama ranch is located about 30 km from Franceville, the main city. The livestock is composed of about 200 animals. N’dama trypanotolerant cattle are reared. The ranch is in a vegetation area composed of savannah and forest transitions.

The rural town of Ossiele is located in the department of Djoué, south of the town of Onga, the capital city of the department. In this region, the vegetation is also made of forest and savannah transitions. In these two regions, the surrounding watercourses are located several kilometers away, making them difficult to access by animals.

The Nyanga ranch is located in the largest savannah zone of the country in the department of Mongo-Moulengui Binza, 65 km from the town Tchibanga (the capital city of Nyanga province) and extends over 100,000 hectares. The Nyanga ranch includes three divisions (Nyanga, Bibora, and Voungou) divided into sections. The study was conducted within the Gala section of Nyanga division 1.

### Animal sampling and blood collection

The samples were collected in cattle, at random (without any distinction in age and sex). Animals that received trypanocide treatment (diminazene aceturate, and two weeks later isometamidium chloride) less than three months before the start of our study were excluded from sampling because the chemoprevention provides a three-month protection period. Approximately 5 mL of whole blood was collected from each animal in ethylenediaminetetraacetic acid (EDTA) vacuum tubes VENOJECT^®^, by puncture of the caudal vein. EDTA tubes containing whole blood were centrifuged at 1300 rpm for 10 min [7] and the plasma was recovered with buffy coat and stored at −20 °C until shipment to the laboratory of the *Centre International de Recherches Médicales de Franceville* (CIRMF), Gabon, for analysis.

### DNA extraction and PCR

Total DNA extraction was conducted from 200 μL of a mixture plasma-buffy coat, using the Qiamp DNA Mini Kit (Qiagen) according to the manufacturer’s guidelines. The DNA was stored at −80 °C until analysis.

Trypanosome research was carried out using two PCRs. In the initial PCR, the intergenic sequence (*ITS1*) primers were used to amplify the partial intergenic sequence region of the ribosomal DNA (rDNA) of trypanosomes [22]. Amplification was performed in 25 μL reaction volumes, containing 2.5 μL of 10X PCR buffer (Platinum^®^ Taq DNA Polymerase, Invitrogen), 2 μL of dNTP (10 μM each), 2 μL MgCl_2_ (50 mM), 0.24 μL of each primer (10 μM), 0.3 μL enzyme Platinum Taq (Platinum^®^ Taq DNA Polymerase, Invitrogen), 12.72 μL of RNAse-free water (Invitrogen), and 5 μL of DNA. Amplification generally involved 5 min at 95 °C followed by 40 cycles of 1 min at 95 °C, 1 min at 60 °C, and 1 min at 72 °C followed by a final elongation at 72 °C for 10 min. The PCR products of approximately 700 base pairs (bp), characteristic of *T. congolense*, were subjected to *T. congolense* subtype-specific PCR to distinguish them between Forest, Savannah, and Kilifi. The primer sets used are shown in Table 1. The PCR conditions are the following for all three PCRs: 5 min at 95 °C followed by 35 cycles including 1 min at 95 °C, 1 min at 55 °C, and 1 min at 72 °C followed by a final elongation at 72 °C for 10 min.

**Table 1.** Primers used for detection of trypanosomes.

Primers	Sequences	Trypanosomes	Size (bp)
ITS 1 CF	5′- CCGGAAGTTCACCGATATTG -3′	*T. congolense*	620–710
ITS1 BR	5′- TTGCTGCGTTCTTCAACGAA -3′	*T. brucei b.*	480
		*T. evansi*	480
		*T. vivax*	250
		*T. simiae*	400
TCF 1	5′-GGACACGCCAGAAGGTACTT-3′	*T. congolense* forest	350
TCF 2	5′-GTTCTCGCACCAAATCCAAC-3′		
TCS 1	5′-CGAGCGAGAACGGGCAC-3′	*T. congolense* savannah	316
TCS 2	5′-GGGACAAACAAATCCCGC-3′		
TCK 1	5′-GTGCCCAAATTTGAAGTGAT-3′	*T. congolense* kilifi	294
TCK 2	5′-ACTCAAAATCGTGCACCTCG-3′		

### Sequence analysis and phylogenetic analysis

Sequence cleaning and assembling of contigs was conducted in ChromasPro 1.5. The nucleotide sequences were compared to those available in the public database using the algorithm “Blastn” of the NCBI BLAST [1]. Phylogenetic analyses were conducted to infer the relationship with other *Trypanosoma* spp. The sequence alignment was performed using the multiple sequence alignment program, ClustalW, contained in MEGA 5 [28]. A neighbor-joining tree was constructed using a Kimura 2-parameter substitution model, with bootstrap values calculated for 1000 replicates. The new sequences were deposited in GenBank with Accession Numbers KX452152–KX452176.

### Statistical analysis

Analyses were performed using R software version 3.1.0 (Development Core Team 2014, USA). The chi-square (χ^2^) test of Pearson was used for the comparison of the proportions of animals infected by different trypanosome species between study areas.

## Results

Twenty-nine rDNA sequences were generated from cattle samples in this study. Blast analysis confirmed that close matches were found with *T. congolense* and *T. vivax*, respectively, for the sequences of 710- and 250-bp products.

**Figure 2.**
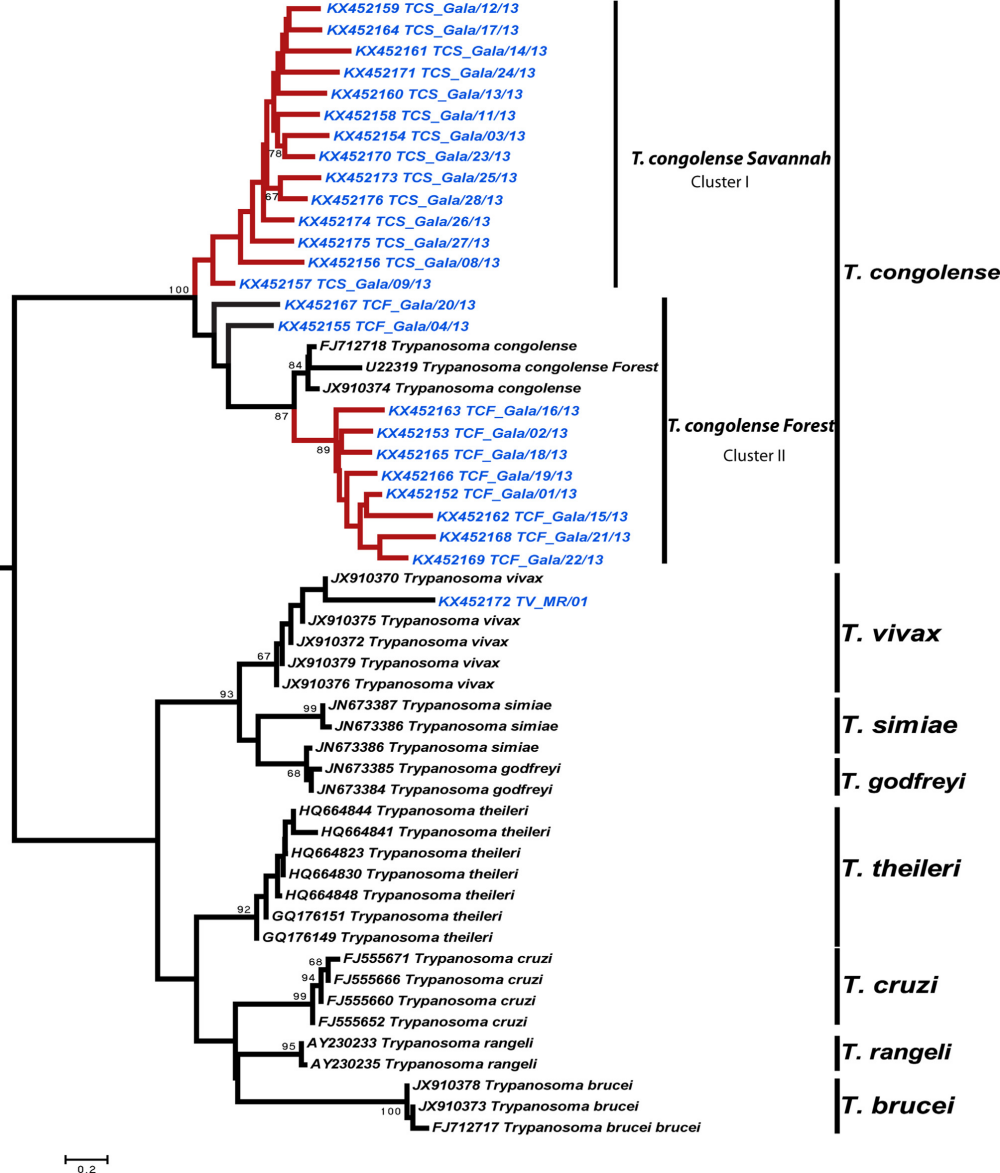
*Phylogenetic tree based on partial ITS1 trypanosome sequence analysis* **.** The tree was visualized with FigTree 1.3.1. Bootstrap values are shown to the left of the branch. Bootstrap values are shown where support is >65%. Sequences generated in this study are shown in blue. Sequences retrieved from GenBank are shown in black with their GenBank accession number.

Twenty-five of 29 sequences generated in this study were included in the phylogenetic analysis. The *T. vivax* sequence obtained from cattle from the Miyama ranch shared 77% identity with the *T. vivax* reference sequence from a cow from Burkina Faso (JX910370), and 69% identity with four other sequences from cows from Burkina Faso (JX910372, JX910375, JX910376, and JX910379). In the phylogenetic tree reconstructions, based on the nucleotide sequences of the *ITS1* region of the ribosomal DNA, the Gabonese *T. congolense* strains from the Gala section grouped into two major clusters. Cluster I comprised 14 *T. congolense* strains belonging to the Savannah subtype; whereas 10 *T. congolense* strains from Gabon and three other strains, including one from Kenya (FJ712718), were grouped within cluster II, bringing together strains belonging to the Forest subtype (Fig. 2). *T. congolense* sequences from cluster I shared 34–76% identity. The *T. congolense* variants grouped within cluster II displayed 43–77% identity with each other at the nucleotide level. These 10 *T. congolense* sequences shared 39–65% identity with the Kenyan reference sequence (FJ712718) and 37–64% with the reference sequence JX910374 from a cow from Burkina Faso.

The screening of 224 samples from N’dama cattle, including 110 from the Gala section, 100 from the Miyama ranch, and 14 from Ossiele, with conventional *ITS1* PCR revealed an overall prevalence of 32.14% (95% CI: 26.03–38.3) (72/224). All the animals sampled appeared healthy and had received treatment against trypanosomosis at least four months before the beginning of the sampling. The infection rates in N’dama from the Gala section, the Miyama ranch, and Ossiele were 57.3% (95% CI: 48.03–66.5) (63/110), 4% (95% CI: 0.2–7.8) (4/100), and 35.7% (95% CI: 10.6–60.8) (5/14), respectively (Table 2). The infection rate of trypanosomosis between the areas showed significant differences (χ^2^ = 68.24, ddl = 2, *p* < 0.001). The overall rates of infection with *T. congolense* and *T. vivax* were 25% (95% CI: 19.3–30.7) and 7.1% (95% CI: 3.8–10.5), respectively (Table 2). Five out of 224 (2.3%) N’dama had mixed infections (*T. congolense*/*T. vivax*). Mixed infections were detected in cattle from Gala. *T. congolense* was the dominant species across the Gala and Ossiele areas, with rates of infection of 47.3% (95% CI: 37.9–56.6) and 28.6% (95% CI: 4.9–52.2), respectively. The rates for *T. vivax* in the same areas were only 10% and 7.1%, respectively. No mixed infections were detected in cattle from Ossiele. According to the chi-squared test, *T. congolense* was significantly more prevalent than *T. vivax* (χ^2^ = 26.48, ddl = 1, *p* < 0.001). Only one trypanosome species was identified in infected animals from the Miyama ranch, namely *T. vivax*, with an infection rate of 4% (Table 2).

**Table 2.** Prevalence of trypanosomosis in study areas and results of molecular characterization of trypanosome species infecting domestic cattle.

Sites	Total	No. total positive ITS1 (%)	Positive *ITS1* PCR (%)		
			TC	TV	TC-TV
Gala	110	63 (57.3)	52 (47.3)	11 (10)	5 (4.5)
Ranch Miyama	100	4 (4)	0	4 (4%)	0
Ossiele	14	5 (35.7)	4 (28.6)	1 (7.1%)	0
Total	224	72 (32.14)	56 (25)	16 (7.1)	5 (2.2)

TC: *Trypanosoma congolense*; TV: *Trypanosoma vivax*; TC-TV: mixed infection with *Trypanosoma congolense* and *Trypanosoma vivax.*

The *T. congolense-*positive samples (*n* = 56) were then screened using a specific PCR for the three subgroups of *T. congolense*: *T. congolense savannah*, *T. congolense forest*, and *T. congolense kilifi*. *T. congolense savannah* was the dominant subgroup with 35 animals infected (62.5%) followed by *T. congolense forest* with 5 infected animals (8.9%). The *T. congolense savannah* subgroup is significantly more predominant (χ^2^ = 35, ddl = 1, *p* < 0.001) than the *T. congolense forest* subgroup in Gala and Ossiele. Furthermore, 16 of 56 (28.6%) animals had mixed infections with both subgroups. No infection with the *T. congolense kilifi* subgroup was discovered.

## Discussion

Prevalence of bovine trypanosomosis and molecular identification of trypanosome species were studied in three cattle farming areas in the south of Gabon, namely the Gala section of the Nyanga ranch, the Miyama ranch, and Ossiele. Phylogenetic analysis was conducted in order to confirm the BLAST results and therefore the identity of the nucleotide sequences obtained. The phylogenetic tree showed that the variants of *T. congolense*, all from the Gala section, displayed considerable genetic variation and were classified into two major clusters. *T. congolense* genetic variants have also been reported in East and West Africa [3, 8]. The identification of this *T. congolense* genetic diversity in the same geographical area indicates the existence of various isolates. As suggested by Auty et al. [3], this genetic diversity cannot be explained only by geographical variation. In the Gala section, cattle are frequently in contact with many other wild animal species, suggesting the possibility of a transfer of strains specific to these wildlife host species [3] through specific species of tsetse. For example, host-specific strains of *T. theileri* were identified in cattle and water buffalo in the same geographical areas [23]. *T. vivax* obtained from cattle from the Miyama ranch shared 69–77% identity with *T. vivax* strains from cows from Burkina Faso. Indeed, domestic ruminants would be likely to share similar parasites. The position of strains KX452167 TCF_Gala/20/13 and KX452155 TCF_Gala04/13 on the phylogenetic tree suggests that they are variants of *T. congolense* forest rather than two hybrid strains between *T. congolense* savannah and *T. congolense* forest. Additional bioinformatics analyses and analyses of recombination events would be necessary.

The molecular prevalence of trypanosomosis was significantly higher in the Gala section than in Ossiele and the Miyama ranch. Out of the 224 N’dama cattle studied, 72 were found PCR positive with trypanosomes, with an overall prevalence of infection of 32.14%. This result was higher than the one found in the cattle in Ghana, using parasitological methods (1.88%) and serology (22.08%) [9], and in the cattle in Northwest Ethiopia using parasitological methods (5.43%) [15]. Otherwise, Nimpaye et al. [21] found an infection rate of 27.08% in domestic animals in Cameroon, using PCR assays. Moreover, Takeet et al. [27] detected 15.1% positive infection with trypanosomes in cattle from Nigeria, using microscopy, while PCR detected 63.7% positive infections. The highest infection rate was found in Gala (57.3%) followed by Ossiele (35.7%) and the Miyama ranch (4%). Indeed, in these three areas, the vegetation is made of savannah and plains crossed by numerous forest galleries and dotted with dense groves. However, the number and proximity of watercourses differ. The Gala section contains several watercourses including rivers and streams. These watercourses, surrounded by moist and shaded forest galleries [29], are shelters for tsetse flies. However, in the Miyama ranch, the watercourses bordering the area are located in distant hilly areas making it difficult to access for animals. The low infection rate found in the cattle from the Miyama ranch could therefore be explained by less frequent contacts between cattle and tsetse flies for the above reasons. Furthermore, the absence of *T. congolense* can probably be due to the absence of a biologically competent vector.

Two species of pathogenic trypanosomes have been identified, namely *T. congolense* and *T. vivax*. These two trypanosome species are the main agents of *nagana* [25]. The presence of these two species in Gabon has been highlighted by an entomological study in the Nyanga valley [29], and in tsetse flies captured during an active outbreak of human trypanosomosis in Komo Mondah [13] and a historic outbreak in Bendjé, in the Ogooué-Maritime Province in western Gabon [11, 12]. In Gala, *T. congolense* and *T. vivax* were identified. These findings support those of Taufflieb [29], who identified these two species in *Glossina palpalis palpalis* in the Nyanga valley with an infection rate of 3% and 2%, respectively. In addition, mixed infections with both trypanosome species have also been identified (4.5%). In the cattle from Ethiopia, *T. congolense* was found to be the dominant species (66.7%), compared to *T. vivax* (9.3%) [22], and 19.4% were mixed infections. According to Taufflieb [29], in the Nyanga area, *G. p. palpalis* is the main tsetse fly vector of trypanosomosis. The *G. p. palpalis* species lives in secondary forest, in vegetation along streams and swamps [31]. In the Miyama ranch, only *T. vivax* was found. This species was already identified by parasitological methods, with an infection rate of 26% in cattle from a ranch located in the south-east of Gabon [14]. The tsetse fly of the *Fusca* group, *G. tabaniformis*, was responsible for the transmission of *T. vivax* in this area [14]. Although a competent vector is present in the area, *T. vivax* can also be found outside of the area of distribution of tsetse because it can be transmitted mechanically by flies from the *Tabanidae*, *Stomoxyinae*, and *Hippoboscidae* families [10, 18].

The overall prevalence of *T. vivax* (7.1%) was lower than that found in cattle and other domestic animals [20, 21, 24, 27]. However, the overall prevalence of *T. congolense* (25%) was high in contrast to that previously reported in other domestic animals [20, 21, 24]. The very low prevalence of *T. vivax* with respect to *T. congolense* would indicate a low transmission of *T. vivax* in the studied localities, which is better controlled by animals because their genetic diversity is more limited than those of *T. congolense* and *T. brucei* [2]. The prevalence of *T. vivax* found in this study using the primers *ITS1* CF and *ITS1* BR is much lower than that obtained by Leak et al. [14] from cattle reared in the same area, using parasitological methods. This difference could be explained, on the one hand, by the improvement over recent years of curative and preventive treatments against trypanosomes, and on the other by the lower sensitivity of *ITS1* CF and BR *ITS1* primers for *T. vivax* compared to species-specific primers [22].

The subtyping of *T. congolense* allowed us to determine the circulation of two subgroups out of three in the Gala section and Ossiele, including *T. congolense savannah* and *T. congolense forest*, respectively, with overall infection rates of 62.5% (57.14% and 5.36% in Gala and Ossiele) and 8.9% (7.14% and 1.79%). Multiple subgroups of *T. congolense* have already been found in multiple ecosystems, and in the same location [16]. Mixed infections with *T. vivax* and *T. congolense* were found. Mixed infections with multiple species of trypanosomes have previously been reported [17]. The infection of cattle with these two pathogenic species exacerbates disease severity even in trypanotolerant cattle. Mixed infections by the two subgroups were also detected (28.6%). In Gabon, the *T. congolense forest* subgroup was predominant in the outbreak of Bendjé, in western Gabon [11]. The infection rate with the *forest* subgroup found in this study is higher than that found in Cameroon, a neighboring country, by Nimpaye et al. [21] in domestic animals. The *savannah* subgroup exhibits a greater virulence than the other two subgroups, experimentally demonstrated in cattle and mice [4, 5].

Further studies are needed to determine the real prevalence of *T. vivax* and the active potential of the infection. Indeed, these findings should be coupled with measurements of packed cell volume or a microscopic confirmation or with another tool, to determine (i) the risk of trypanosomosis in the main breeding areas of this country in Central Africa, because of the co-circulation of these two pathogenic species of trypanosomes, and (ii) the need to increase control measures for trypanosomosis in livestock breeding areas in Gabon, because although trypanotolerant, N’dama cattle could be a reservoir for these two parasites (*T. vivax* and *T. congolense*) and therefore a risk for programs targeting the elimination of animal trypanosomosis in the region and an obstacle to the introduction of other more trypanosusceptible but productive cattle breeds. On the other hand, these trypanotolerant cattle can be reared in such tsetse infested areas. This is an enormous advantage compared to other breeds, and it shows that they represent a key in biodiversity which has to be promoted.

## Conflict of interest

The authors declare that they have no conflict of interest.
